# Using the “target constituent removal combined with bioactivity assay” strategy to investigate the optimum arecoline content in charred areca nut

**DOI:** 10.1038/srep40278

**Published:** 2017-01-05

**Authors:** Wei Peng, Yu-Jie Liu, Mei-Bian Hu, Dan Yan, Yong-Xiang Gao, Chun-Jie Wu

**Affiliations:** 1 College of Pharmacy, Chengdu University of Traditional Chinese Medicine, Chengdu 610075, P.R. China; 2 College of Basic Medicine, Chengdu University of Traditional Chinese Medicine, Chengdu 610075, P.R. China

## Abstract

Charred areca nut (CAN) is used to treat dyspepsia and abdominal distension in children. However, reports revealed that arecoline, the most important active constituent of CAN, possesses potential toxicities. This study was designed to investigate the optimum arecoline content in CAN, using the “target constituent removal combined with bioactivity assay” strategy. Based on PTLC method, we prepared CAN lacking all arecoline (WAC-100R) and a series of different ratios of arecoline-removed CAN samples (WAC-Rx). MTT and acute toxicity assays indicated that decreasing content by 50% decreased CAN toxicity significantly. Animal results revealed arecoline contents over 50% could guarantee the beneficial effects of CAN on gastrointestinal tract. Additionally, decreasing arecoline content in CAN by 50% decreased its pro-apoptotic effects significantly. Furthermore, decreasing 50% arecoline content in CAN down-regulated the expressions of Cleaved-Caspase-3, c-*jun*, c-*fos*, COX-2, PGE2, and IL-1α. Thus, our results revealed that CAN with 50% arecoline content (WAC-50R) has similar beneficial effects on the gastrointestinal tract to CAN, whereas its toxicity was decreased significantly. Collectively, our study suggested that the strategy of “target constituent removal combined with bioactivity assay” is a promising method to identify the optimum arecoline content in CAN, which is approximately 0.12%.

Charred areca nut (CAN), also called Arecae Semen Tostum in the Chinese Pharmacopoeia (CH.P), is the processed product of the fruits of *Areca catechu* (Arecaceae)^1^,^2^. In addition, CAN is one of the most commonly used traditional Chinese medicines (TCMs). CAN is considered as a significant herbal drug to treat dyspepsia and abdominal distension in children^3^. Currently, dozens of Chinese TCM formulas to treating childhood diseases contain CAN, such as *Bing Lang Si Xiao* tablets, *Fei Er* pills, *Jian Wei Xiao Shi* tablets, and *Si Mo* decoction^2^. Previous reports revealed that the main active constituents in CAN are alkaloids, and in particular, arecoline is the most important alkaloid in this herbal medicine^2^,^4^. For thousands of years, CAN was regarded as a safe agent in China^5^. However, recently, there have been numerous toxicological reports demonstrating that arecoline in CAN has potential toxicities, inducing oral submucosal fibrosis (OSF) and carcinogensis^6^,^7^. However, arecoline is also reported to be the most important active constituent, providing beneficial effects on the digestive system^8^,^9^. Therefore, it is urgent and important to find the optimum arecoline content in CAN that could balance the pharmacological and toxic effects.

Recently, there have been reports of a new strategy to screen active compounds from herbal medicines by removing or adding constituents^10^,^11^,^12^,^13^. Yan *et al*.^10^ indicated that they obtained inspiration from functional genetic methods. They removed and added individual constituents to the *Rhizoma coptidis* (a famous herbal medicine) combined with a bioactivity assay, which demonstrated that berberine and coptisine were the main antibacterial constituents of *R. coptidis*. The authors suggested that the method comprising removing and adding constituents combined with a bioassay represents a high-throughput screening assay, and could be a useful strategy to screen active constituents in herbal medicines. We hypothesized that this strategy could be also used to investigate the optimum contents of those constituents in herbal medicines that are both toxic and bioactive. We believe that removing toxic constituents combined with a bioassay might be useful to decrease the toxicity of a toxic herbal medicine, achieving “toxicity-reducing and efficacy-maintaining” effects. We believe that this novel strategy will enhance the therapeutic effect and safety of herbal medicines in the future. Thus, the present study investigated the optimum arecoline content in CAN using the “target constituent removal combined with a bioactivity assay” strategy, which would be beneficial for the safe use of this herbal medicine to treat diseases in children. The experimental strategy used in the present investigation is described in Fig. 1.

## Results

### Results of the Preparation of WAC-Rx samples

**Figure 2:**
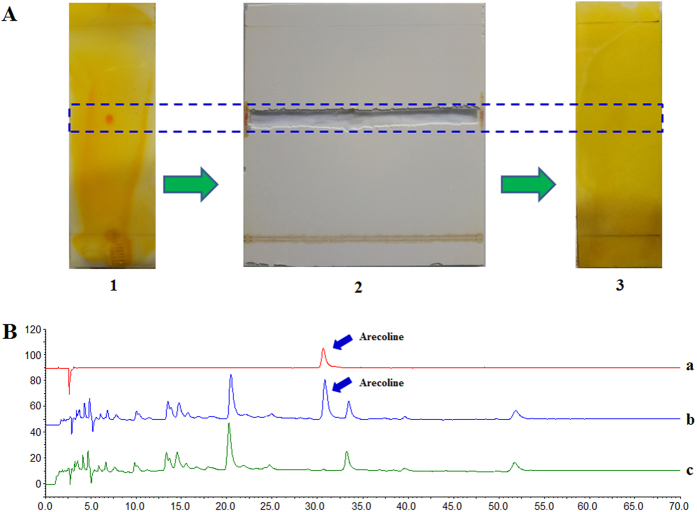
Preparation of the arecoline removed totally charred areca nut samples (WAC-100R). (**A**) Preparation of the WAC-100R samples by preparative thin layer chromatography (PTLC). **1** represents the WAC samples and the red band represented the arecoline, **2** shows the removal the arecoline band by PTLC, **3** shows the WAC-100R samples. (**B**) High performance liquid chromatography (HPLC) assay to determine the arecoline content of the WAC-100R samples. a–c represented the arecoline, WAC and WAC-100R, respectively.

As shown in Fig. 2A, the arecoline band could not be detected by preparative thin-layer chromatography (PTLC). In addition, HPLC analysis (Fig. 2B) also demonstrated that the arecoline was almost completely removed from the water extracts of charred areca nut (WAC) samples. Therefore, the sample obtained after PTLC was treated as the arecoline totally removed WAC sample (WAC-100R, “R” means “removal”) and used in subsequent experiments. A series of WAC samples with different ratios of arecoline removal (WAC-Rx), including WAC-90R, WAC-80R, WAC-70R, WAC-60R, WAC-50R, WAC-40R, WAC-30R, WAC-20R, and WAC-10R, were also prepared based on WAC-100R and WAC (Table 1).

### Cytotoxicity of the different WAC-Rx samples

As shown in Table 2, the cytotoxicity of WAC decreased notably when the arecoline contents were reduced. WAC possessed the strongest cytotoxicity on HaCaT cells after a long treatment time (72 h), with an IC_50_ value of 107.73 μg/mL. Interestingly, when the arecoline was reduced by over 50% compared with WAC, the IC_50_ values of WAC-Rx became very high. From the WAC-50R downwards, no obvious inhibitory effects on proliferation were observed, and their IC_50_ values were all over 400 μg/mL. These results indicated that decreasing the arecoline content by more than 50% might be beneficial for the safe use of CAN.

### Acute toxicity the different WAC-Rx samples in mice

The various WAC preparations were administered to mice and the mice were observed for 7 days. During this period, the mice in WAC-30R and WAC groups were become motionless, and displayed piloerection, breathlessness and body writhing. However, no obvious toxic effects were observed in the mice in the WAC-50R and WAC-100R groups. In addition, the LD_50_ value of WAC was calculated as 15.302 g/kg, and the 95% confidence intervals were between 13.975 and 16.754 g/kg. For the other three tested samples (WAC-30R, WAC-50R and WAC-100R), no deaths were observed using any of the test doses (6.554, 8.192, 10.24, 12.8, 16.0, and 20.0 g/kg). These results indicated that decreasing the arecoline content by over 50% could enhance the safety of CAN.

### Effects on gastrointestinal smooth muscle contractility of the different WAC-Rx samples

**Figure 3:**
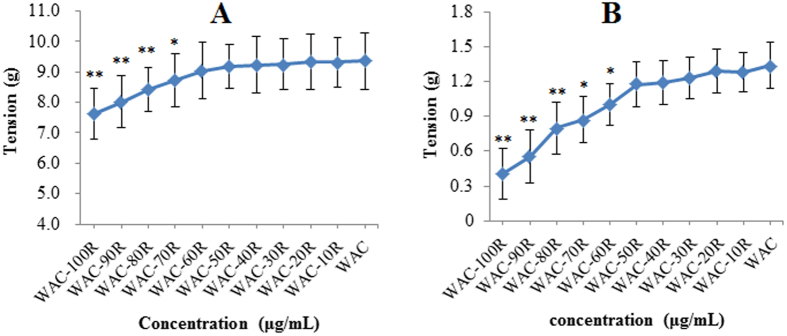
Effects of different WAC-Rx samples on the contractility of stomach (**A**) and duodenum (**B**). The concentration of all the test samples was 40 μg/mL; **p < 0.01, *p < 0.05 *vs*. WAC.

According to previous research^14^,^15^, areca nut water extracts exhibit their best gastrointestinal smooth muscle contractility at 40 μg/mL, therefore the final concentrations of the WAC-Rx samples were set at 40 μg/mL for the further explorations. Figure 3 shows that compared with WAC, the effects of WAC-100R (*p* < 0.01), WAC-90R (*p* < 0.01), WAC-80R (*p* < 0.05), and WAC-70R (*p* < 0.05) on stomach and duodenum strips were significantly decreased. In addition, with increasing arecoline content, the stomach and duodenum contractility increased gradually. Importantly, WAC-50R showed almost the same stomach and duodenum contractility as WAC. These results indicated that arecoline at 50% or above could guarantee the effects of CAN.

### Results of the gastric emptying and small intestinal transit experiments in mice

**Figure 4:**
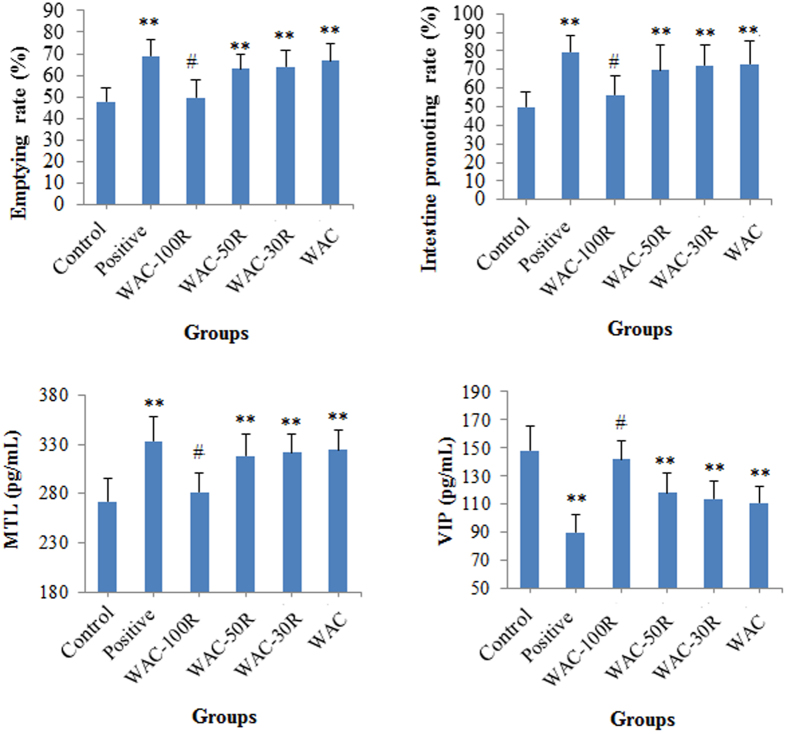
Effects of WAC-Rx on gastric emptying, intestine promotion, and contents of motilin (MTL) and vasoactive intestinal peptide (VIP) in mice. Mosapride (30 mg/kg) was used as the positive control drug. **p < 0.01, *p < 0.05 *vs.* control; ^#^*p* < 0.05 *vs.* WAC.

The results shown in Fig. 4 show that preserving 50% of the arecoline content in WAC caused significant gastric emptying and intestine promoting effects (*p* < 0.01), which showed no obvious difference with those of the WAC samples (*p* > 0.05). In addition, our results also revealed that preserving 50% of the arecoline content in WAC significantly increased the serum content of motilin (MTL) (*p* < 0.01), whereas it decreased the vasoactive intestinal peptide (VIP) content in serum (*p* < 0.01). No obvious difference was found between WAC-50R and WAC (*p* > 0.05).

**Figure 5:**
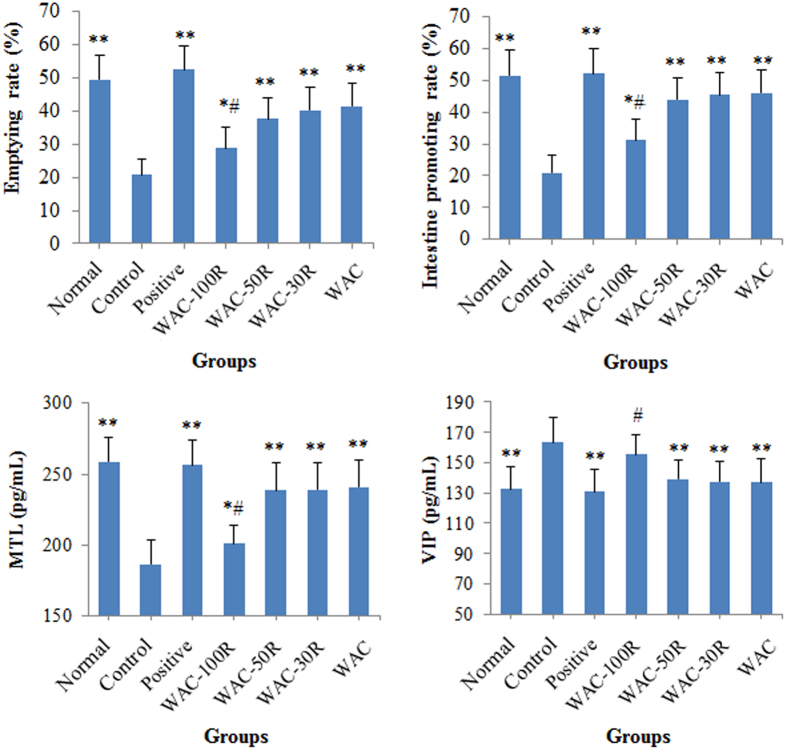
Effects of WAC-Rx on gastric emptying, intestine promotion, motilin (MTL) and vasoactive intestinal peptide (VIP) contents in atropine sulfate-treated mice. Mosapride (30 mg/kg) was used as the positive control drug. **p < 0.01, *p < 0.05 *vs.* control; ^#^*p* < 0.05 *vs.* WAC

Furthermore, a similar result was obtained for the atropine sulfate treated mice model (Fig. 5). WAC-50R, WAC-30R, and WAC possessed obvious gastric emptying and intestine promoting effects (*p* < 0.01), and showed no obvious difference compared with WAC (*p* > 0.05). WAC-50R, WAC-30R, and WAC could increase the MTL content (*p* < 0.01) and decreased the VIP content in serum (*p* < 0.01). No difference was observed between the 50% arecoline preserved WAC-Rx sample and WAC (*p* > 0.05). These results revealed samples that retaining over 50% arecoline contents in WAC had similar effects to those of WAC.

### Pro-apoptotic effect of different WAC-Rx samples on HaCaT cells

**Figure 6:**
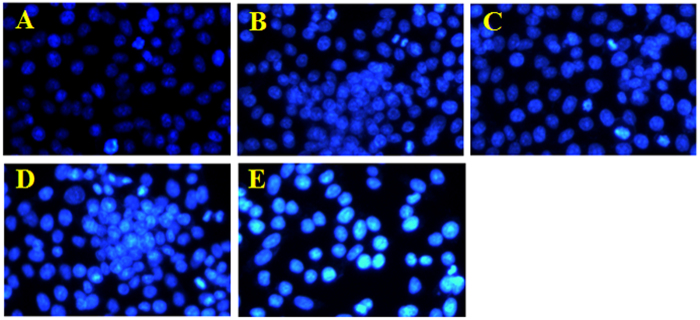
Pro-apoptotic effect of different WAC-Rx samples on HaCaT cells by 4′,6-diamidino-2-phenylindole (DAPI) staining. HaCaT cells were treated with WAC-Nx at 300 μg/mL for 72 h, and the apoptotic cells were detected by DAPI staining and visualized under a fluorescent microscope (×200). (**A**–**E)** represented the control, WAC-100R, WAC-50R, WAC-30R, and WAC, respectively.

**Figure 7:**
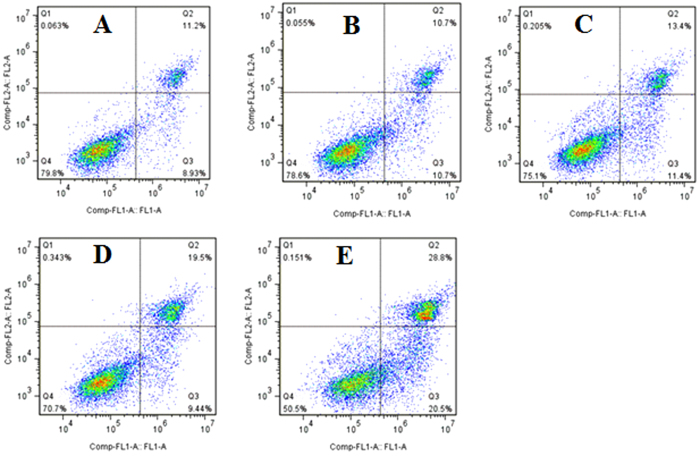
Pro-apoptotic effect of different WAC-Rx samples on HaCaT cells by flow cytometry. HaCaT cells were treated with WAC-Rx at 300 μg/mL for 72 h, and the apoptotic cells were detected by staining with AnnexinV-fluorescein isothiocyanate (FITC)/propidium iodide (PI) followed by flow cytometry analysis. (**A**–**E)** represented the control, WAC-100R, WAC-50R, WAC-30R and WAC, respectively.

To determine whether decreasing the arecoline content could reduce the pro-apoptotic effects of WAC on HaCaT cells, the cells were stained with 4′,6-diamidino-2-phenylindole (DAPI) to examine nuclear morphological changes (Fig. 6). In the control group, the cell nucleus was round and intact, with faint DAPI staining, indicating that the cells were alive. However, after treatment with WAC at 300 μg/mL for 72 h, we observed the characteristic apoptotic features in HaCaT cells, including marked nuclear condensation and bright staining with DAPI. However, no obvious apoptosis was observed in the HaCaT cells treated with WAC-50R and WAC-100R. After treatment with WAC-Rx for 72 h, HaCaT cells were stained with AnnexinV-fluorescein isothiocyanate (FITC)/propidium iodide (PI) and detected by flow cytometry analysis; similar results to those of DAPI staining were obtained (Fig. 7). These results also demonstrated that decreasing the arecoline contents in CAN by over 50% could decrease its pro-apoptotic effects significantly.

### Effects of different WAC-Rx samples on the expressions of cleaved-caspase-3 (C-caspase-3), c-jun, c-fos, COX-2, PGE2, and IL-1α

**Figure 8:**
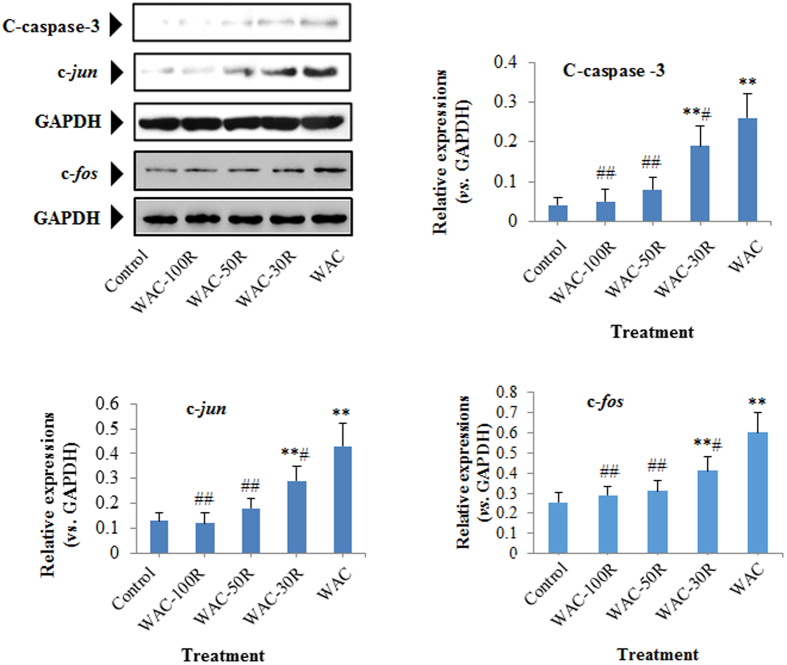
Effect of different WAC-Rx samples on the expressions of C-caspase-3, c-*jun*, and c*-fos* in HaCaT cells. HaCaT cells were treated with WAC-Rx at 300 μg/mL for 72 h, and the expressions of C-caspase-3, c-*jun*, and c-*fos* in HaCaT cells were detected by western blotting. **p < 0.01, *p < 0.05 *vs.* control; ^##^*p* < 0.01, ^#^*p* < 0.05 *vs.* WAC.

**Figure 9:**
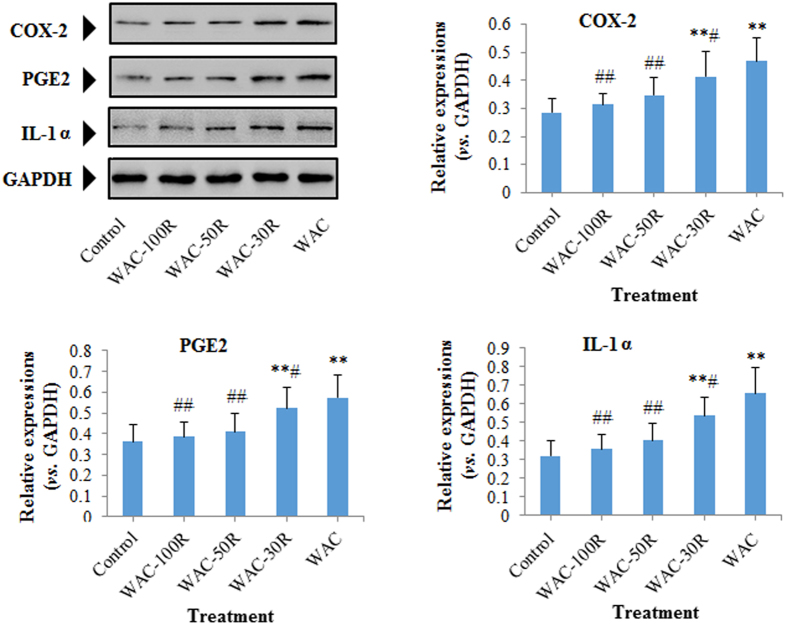
Effect of different WAC-Rx samples on the expressions of COX-2, PGE2, and IL-1α in HaCaT cells. HaCaT cells were treated with WAC-Rx at 300 μg/mL for 72 h, and the expressions of COX-2, PGE2, and IL-1α in HaCaT cells were detected by western blotting. **p < 0.01, *p < 0.05 *vs.* control; ^##^*p* < 0.01, ^#^*p* < 0.05 *vs.* WAC.

We determined the protein levels of cleaved-caspase-3 (C-caspase-3), c-*jun*, c-*fos*, COX-2, PGE2, and IL-1α in HaCaT cells using western blotting. Figures 8 and 9 showed that the expressions of C-caspase-3, c-*jun*, c-*fos*, COX-2, PGE2, and IL-1α in HaCaT cells were downregulated gradually with decreasing arecoline content. Interestingly, similar to the WAC-100R, no obvious difference was observed between WAC-50R and the control group (*p* > 0.05).

## Discussion

Herbal medicines are known to be effective in treating various diseases; however, the constituents of herbal medicines are very complex. In addition, compared with chemical drugs, herbal medicines commonly treat diseases via multiple targets and via the synergistic effects of multiple components instead of one constituent^16^. Furthermore, there are both therapeutic and toxic agents in the herbal medicines, and some toxic constituents in herbal medicines are also the main active constituents^17^,^18^. Therefore, it is essential to find the optimum contents of toxic constituents in herbal medicines to achieve “toxicity-reducing and efficacy-maintaining” effects.

Removing constituents can be performed by preparative chromatographic techniques, including PTLC, preparative column chromatography, and preparative high performance liquid chromatography (PHPLC)^10^,^11^,^12^,^13^. Thereafter, *in vivo* and *in vitro* bioassays are used to screen the change in bioactivity. Based on our preliminary experimental results (Supplementary files Figures S1 and S2) and related references^19^,^20^, arecoline is the main alkaloid among the total alkaloids of CAN extracted by CHCl_3_. Importantly, we also found that there was no other obvious constituent that interfered in the same position as the arecoline band (Figure S2). Therefore, we believed that PTLC could remove the arecoline effectively to prepare the WAC-100R sample. However, compared with PTLC, PHPLC might be more efficient and feasible, thus we hypothesized that our present strategy would be optimized using PHPLC technology.

In previous studies, the HaCaT cell line was used extensively as a substitute for oral keratinocytes or oral epithelial cells to investigate oral diseases^7^,^21^. Therefore, in our present investigation, we selected this cell model to investigate the cytotoxicities of the WAC-Rx samples. Previous research showed that areca nut extracts and arecoline are cytotoxic to normal human cells, including epithelial cells, endothelial cells, hepatocytes, splenocytes, myoblasts, and lymphocytes^2^,^22^,^23^,^24^. Our results also showed that CAN extracts (WAC) possessed cytotoxic effects on HaCaT cells (IC_50_ = 107.73 μg/mL). Interestingly, after removing 50% of the arecoline, the cytotoxicity of the CAN extracts decreased sharply. It was reported that the water extracts of areca nut are toxic to mice^3^. Our results showed that LD_50_ value of water extracts of CAN was 15.302 g/kg (oral administration). However, after removing half of the arecoline content, the toxicity decreased sharply, and no obvious toxic effect was observed. Treating dyspepsia and abdominal distension in children are the most important functions of CAN. CAN is believed to promote contractility of the digestive tract smooth muscle^2^,^8^. Therefore, we selected the gastrointestinal promoting effects to evaluate the bioactivities of WAC-Rx samples. In addition, we also determined the serum contents of MTL and VIP, which are two important regulatory factors of the digestive function. MTL, a polypeptide secreted by Mo cells, induces strong gastrointestinal motility. By contrast, VIP, released from the inhibitory vasoactive intestinal peptidergic fibers, leads to inhibition of gastric acid secretion and relaxation of the gastrointestinal tract^25^,^26^,^27^,^28^. Thus, increasing MTL and decreasing VIP could promote gastrointestinal motility. Our results suggested that preserving 50% or more of the arecoline content could guarantee the effects of the CAN extracts.

It has been reported that two of the most important toxicities of areca nut or arecoline are oral submucosal fibrosis (OSF) and carcinogensis^7^,^29^,^30^,^31^. Previous reports indicated that areca nut and arecoline could induce apoptosis and oxidative stress, and upregulate proto-oncogenes (c-*jun* and c-*fos*) and inflammation related proteins^6^,^16^,^32^. As the most important effector molecules of apoptosis, caspase-3 is a key executioner of apoptosis, and is cleaved during apoptosis. Previous reports indicated that the expression level of cleaved caspase-3 (C-caspase-3) could reflect the extent of apoptosis^33^,^34^ Areca nut extracts and arecoline could induce obvious apoptosis in human epithelial cells (HaCaT cells)^7^, C2C12 myoblast cells^23^,^35^, and hepatocytes (Clone-9 cells)^22^,^36^. The present results showed that decreasing the arecoline content by more than 50% decreased the number of apoptotic cells and down-regulated the C-caspase-3 expression in HaCaT cells. These results suggested that decreasing the arecoline content in CAN by over 50% could decrease the risk of OSF.

Over-expressions of c-*jun* and c-*fos* are implicated in a broad spectrum of malignancies. Similar to c-jun, c-*fos* is expressed at a low level in normal cells; however, its overexpression might result in malignancies^36^,^37^,^38^. Increasing evidence has demonstrated that inflammation could encourage tumor development^39^,^40^. COX-2 is a curial enzyme for the induction of the inflammatory reaction and PGE2 and IL-1 are two important inflammatory mediators. Previous reports revealed that upregulation of COX-2 and PGE2 could result in cell proliferation, angiogenesis, and metastasis of malignancies^32^,^41^,^42^,^43^. Moreover, COX-2-derived PGE2 is important in tumors that evade immune surveillance by re-educating infiltrating inflammatory/immune cells during tumorigenesis^32^,^44^. IL-1 could induce the release of COX-2 and PGE2, leading to the inflammatory cascade. Previous research also indicated that prolonged upregulation of IL-1 might induce tumorigenesis and malignant cell transformation^45^,^46^. In addition, over-expression of COX-2-derived PGE2 and IL-1α have been observed clinically in various cancers^32^,^47^,^48^. Thus, the downregulation of COX-2, PGE2, and IL-1 would reduce the risk of carcinogenesis. Previous research into the areca nut indicated that areca nut extracts could significantly increase the expressions of COX-2, PGE2, and IL-1α in human immune cells and HaCaT cells^6^,^21^,^32^. In the present study, we found that decreasing the arecoline content to 50% (WAC-50R) significantly downregulated the expressions of c-*jun* c-*fos*, COX-2, PGE2, and IL-1α in HaCaT cells. These results demonstrated that removing 50% of the arecoline in areca nut could decrease the risk of carcinogenesis from areca nuts significantly.

Our results showed that the arecoline content in the CAN was 0.239% (Supplementary files Figure S3). Interestingly, after removing approximately 50% of the arecoline, the CAN retained its equal gastrointestinal promoting effects, whereas its toxicities decreased significantly. Thus, based on our results, we proposed that the optimum content of arecoline in CAN is approximately 0.12%.

In conclusion, we demonstrated that the strategy of “target constituent removal combined with a bioactivity assay” was suitable to determine the optimum arecoline content of CAN. Thus, we suggest that this strategy is a promising method to investigate other toxic constituents with activities in herbal medicine.

## Materials and Methods

### Plant material

The charred areca nuts were purchased from the Neautus Chinese Herbal Pieces Ltd. Co. (Chengdu, China), and were identified by Prof. Chun-Jie Wu (College of Pharmacy, the Chengdu University of Traditional Chinese Medicine). The average arecoline contents of the CAN used in our present study was 0.239% (as determined by high performance liquid chromatography (HPLC)) (Supplementary files Figure S1 and Table S1). A voucher specimen (S-20141116) was deposited in our laboratory.

### Animals

Experimental groups consisted of SD rats (200 ± 20 g), which were purchased from the Dashuo Experimental Animal Center (Chengdu, China). They were housed at 21 ± 1 °C under a 12 h light/dark cycle and had free access to standard pellet diet (Purina chow) and tap water. All animal treatments were performed strictly in accordance with international ethical guidelines and the National Institutes of Health Guide concerning the Care and Use of Laboratory Animals. The experiments were carried out with the approval of the Animal Experimentation Ethics Committee of Chengdu University of Traditional Chinese Medicine.

### Chemicals

Dimethyl sulfoxide (DMSO), 4′,6-diamidino-2-phenylindole (DAPI) and 3-(4,5-dimethylthiazol-2-yl)-2,5-diphenyltetrazolium bromide (MTT) were purchased from Sigma (MO, USA). The DMEM media and fetal bovine serum (FBS) were purchased from Invitrogen (Carlsbad, California, CA, USA). The Annexin V-FITC/PI kit was purchased from BD Bioscience (San Diego, CA, USA). Preparative silica-gel G TLC plates were purchased from Qingdao Haiyang Chemical Co. (Qingdao, China). The IP Cell Lysis Buffer, BCA Protein Assay Kit, QuickBlock™ blocking buffer, Beyo ECL Star, and HRP-conjugated secondary antibody were purchased from the Beyotime Institute of Biotechnology (Haimen, China). Primary antibodies for c-*jun*, c-*fos*, COX-2, PGE2, C-caspase-3, and GAPDH were purchased from the Abcam Co. (Cambridge, UK). The primary anti-body for IL-1α was purchased from the Santa Cruz Biotech (Tokay, Japan). The arecoline standard reference was purchased from the National Institute for the Control of Pharmaceutical and Biological Products (Beijing, China). Injectable atropine sulfate was purchased from the Sichuan Medco Huakang Pharmcetutical Co. (Chengdu, China). Mosapride dispersible tablets were purchased from the Kanghong Pharmceutical Co. (Chengdu, China). MTL and VIP ELISA kits were purchased from the Wuhan USCN Life Science Inc. (Wuhan, China). All other chemicals used in this study were of analytical reagent grade.

### Preparation of charred areca nut samples with all arecoline removed

**Figure 10:**
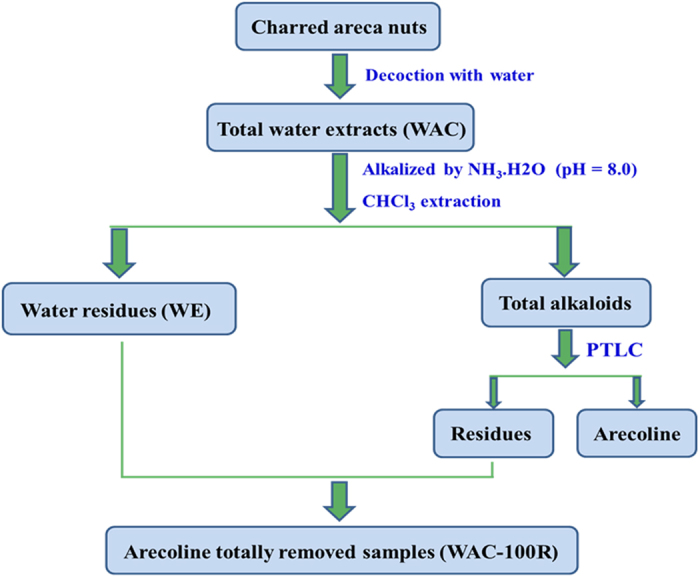
Flow chart of the preparation of CAN with arecoline totally removed (WAC-100R).

The CAN sample with arecoline totally removed (WAC-100R) was prepared using the preparative thin layer chromatography (PTLC) method, which was detailed in a previous study^10^,^11^. Briefly, the CAN was powdered and subsequently extracted by decocting with water. Then, the extracts (WAC) were filtered and concentrated *in vacuum* under 60 °C. The concentrated water extracts of CAN were dissolved in water and re-adjusted with NH_3_.H_2_O to approximately 8.0, and subsequently extracted with trichloromethane (CHCl_3_). Then, the water part (WE) was concentrated under a vacuum, and CHCl_3_ was recovered in a vacuum under 60 °C. The residue was treated as the total alkaloids. Subsequently, the PTLC method was used to separate arecoline from the total alkaloids of CAN. In this study, 20 × 20 cm preparative TLC plates were used, the mobile phase was *n*-hexane: ethyl acetate: acetone: NH_3_.H_2_O (10:7:3:0.2), and the improved bismuth potassium iodide reagents were used to detect the arecoline bands (red). After the arecoline bands were removed, all the residual silica gel of the PTLC plate was collected, washed with acetone, and filtered. The filtrates were then concentrated in a vacuum. Finally, the residues of the total alkaloids were re-added to the WE, the mixture was dried *in vacuum* under 60 °C to produce the CAN samples with arecoline totally removed (WAC-100R) (Fig. 10). HPLC analysis was used to evaluate the contents of arecoline in WAC-100R, which was performed on an Agilent 1260 HPLC system with a CAPCELL PAK MG II S5 C_18_ chromatographic column (250 mm × 4.6 mm, i.d. 5 μm, Shiseido, Japan) at 230 nm, the sample injection volume was 10 μL, and the column temperature was 30 °C. Separation was performed using gradient elution [acetonitrile (A) /0.1% aqueous formic acid (containing 0.1% sodium heptanesulfonate, B)] gradient at a flow rate of 1 mL/min. Samples were analyzed using a gradient program as follows: 0–10 min, 92% B; 10–40 min, 92–86% B; 40–70 min, 86–75% B. Furthermore, a series of WAC samples with different ratios of arecoline removal (WAC-Rx) (Table 2) was prepared, including WAC-90R, WAC-80R, WAC-70R, WAC-60R, WAC-50R, WAC-40R, WAC-30R, WAC-20R, and WAC-10R.

### Cell culture

A cell line comprising spontaneously immortalized human normal dermal keratinocyte cells (HaCaT) was purchased from the American Type Culture Collection (MD, USA). The cells were cultured in DMEM medium supplemented with 10% FBS and antibiotics (100 U/mL penicillin and 100 U/mL streptomycin). The cell lines were cultured at 37 °C in 5% CO_2_/95% air.

### Cell cytotoxicity assay

Cell viability was performed using an MTT assay according to a previously reported method^21^,^49^. Briefly, cells (5000 cells/well) were plated and cultured in 96-well plates for 4 h, and subsequently treated with WAC or WAC-Rx at a series of concentrations (3.125, 6.25, 12.5, 25, 50, 100, 200, and 400 μg/mL) for 72 h. Then, MTT assay was carried out to assay cell proliferation inhibition (%) (n = 4) by detecting the optical density (OD) at 570 nm. Then, the half maximal inhibitory concentration (IC_50_) value was calculated. The inhibition rate was calculated using the following formula: (OD_control_ − OD_treatment_)/OD_control_ × 100%.

### Acute toxicity assay

To evaluate the *in vivo* toxicities of different WAC-Rx samples (including WAC-100R, WAC-50R, WAC-30R, and WAC), an acute toxicity assay in mice was carried out according to a previous method^1^. Briefly, for each test sample, there were six dosage groups, each comprising 10 mice (n = 10). The testing dosages were 6.554, 8.192, 10.24, 12.8, 16.0, and 20.0 g/kg, respectively (the dose-ratio between groups was 0.8), and the corresponding logarithmic doses were 0.8, 0.9, 1.0, 1.1, 1.2, and 1.3, respectively. Test samples were administered orally, and the mortality rates of the mice within a 7-day period were observed and recorded. The acute toxicities of the test samples were evaluated by the LD_50_ value calculated by the Karber’s method^50^.

### Tissue preparation and determination of the gastrointestinal smooth muscle contraction

The present study was carried out according to a previously reported method^51^. Gastrointestinal smooth muscle strips (stomach and duodenum) were isolated from normal SD rats and cut into approximately 2.0 cm segments. Gastrointestinal smooth muscle contractility was determined using a four-channel automatic organ bath system (Panlab S.L., Spain). One end of the duodenum segment was fixed to the bottom side of the tissue bath chamber (20.0 mL volume), and the other end was connected to a force-displacement transducer in the longitudinal direction. The resting tension was set at 2.0 g and the buffer was changed every 10 min until a dynamically equilibrated contractile state of the duodenum segment was obtained. The contractile response was recorded using the physiological recording system of Panlab (LabChart 7 in Chinese, Spain). The mean amplitude was calculated from eight independent assays.

### Gastric emptying and small intestinal transit experiments in normal mice and atropine sulfate-treated mice

Gastric emptying and small intestinal transit assays were carried out according to the method reported previously, with minor modifications^29^,^51^. Mice were divided randomly into six groups (n=10) after fasting for 18 h: Control, positive, WAC-100R, WAC-50R, WAC-30R, and WAC. All the WAC samples (200 mg/kg/d) and positive control drugs (Mosapride, 30 mg/kg/d) were administered orally for 3 days, and mice in the control group were treated with (distilled water, 10 mL/kg/d). Within 30 min of the last WAC sample administration, all the mice were administered orally a test meal (containing active carbon, CMC-Na, milk, glucose, and starch, 0.3 g/mice). Subsequently, 20 min after the test meal administration, blood samples were collected from the aorta abdominalis, and then the mice were sacrificed by cervical dislocation. The stomach was removed after ligation of the pylorus and preventriculus. The total stomach weights were measured (W_n_), and subsequently the pure stomach weights were measured after removing the stomach contents (W_0_). The gastric emptying rate (%) was calculated according to the following equation (1):


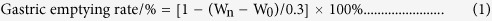


In addition, the small intestine was separated, and the total length of the pylorus to the ileocaecal junction (L_0_), and the distance traveled of the active carbon (L_n_), were measured. The intestinal propulsion rate (%) was calculated according to the following equation (2):


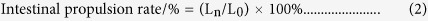


After serum samples were prepared by centrifugation 15 min (1800 × *g*), the MTL and VIP levels in serum were determined following the protocol of commercial ELISA kits.

Furthermore, mice with gastrointestinal motility disorders were constructed by the subcutaneous injection of atropine sulfate (2 mg/kg). Gastric emptying and small intestinal transit experiments were then carried out according to the methods mentioned above.

### Nuclear staining with DAPI

DAPI staining assay was carried out according to a previously reported method^52^. Briefly, cells were exposed to different WAC-Rx (WAC-100R, WAC-60R, WAC-30R, and WAC) at 300 μg/mL for 72 h. The cells were then stained with DAPI, and the changes in the cells’ nuclei were determined and photographed using a fluorescence microscope (Olympus, BX-60, Tokyo, Japan).

### Apoptosis assay by flow cytometer

In addition, apoptotic cells were also determined by staining with FITC conjugated Annexin V/PI by flow cytometry, according to the commercial kit’s instructions. Annexin V-positivity and PI-negativity indicated percentage of cells in early apoptosis, while the percentage of cells in late apoptosis was calculated by Annexin V-positivity and PI-positivity^53^.

### Western blotting assay

HaCaT cells (2.0 × 10^4^/mL) were seeded in 6 cm culture dish and incubated with different WAC-Rx samples (300 μg/mL) for 72 h. The cells were then collected and washed with phosphate buffered saline (PBS) three times, and total cell proteins were extracted using IP Cell Lysis Buffer. The protein concentration was measured by using a BCA Protein Assay Kit. Subsequently, 30 μg of proteins were separated by 10% sodium dodecyl sulfate-polyacrylamide gel electrophoresis (SDS-PAGE) and blotted onto polyvinylidene fluoride (PVDF) membranes (EMD Millipore, Boston, MA, USA). After blocking with QuickBlock™ blocking buffer (TBS) for 2 h, the PVDF membranes were washed with TBST three times. The PVDF membranes were then incubated with specific primary antibodies, washed, and incubated with HRP-conjugated secondary antibodies. Finally, the proteins bands were visualized using Beyo ECL Star. Immunoblotting signals were scanned and determined quantitatively using a ChemiDoc XRS gel imaging system (Bio-Rad, Hercules, CA, USA) and Quantity One software, respectively.

### Statistical analysis

All Data were expressed as mean ± standard deviation (SD). Data were analyzed by Student’s t-test or ANOVA using the SPSS software (SPSS for Windows 18.0, SPSS Inc., USA). *P*-values less than 0.05 were considered statistically significant.

## Additional Information

**How to cite this article:** Peng, W. *et al*. Using the “target constituent removal combined with bioactivity assay” strategy to investigate the optimum arecoline content in charred areca nut. *Sci. Rep.* **7**, 40278; doi: 10.1038/srep40278 (2017).

**Publisher's note:** Springer Nature remains neutral with regard to jurisdictional claims in published maps and institutional affiliations.

## Supplementary Material

Supplementary Files

## Figures and Tables

**Figure 1:**
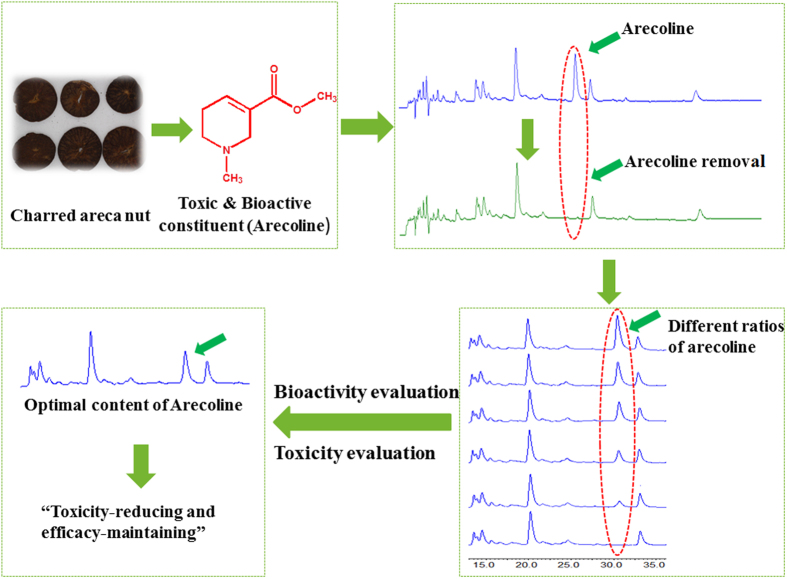
The strategy used to investigate the optimum arecoline content in charred areca nut.

**Table 1:** Composition of the series different ratios of arecoline-removed charred areca nut samples (WAC-Rx).

	WAC-100R	WAC-90R	WAC-80R	WAC-70RN	WAC-60R	WAC-50R
WAC-100R	100%	90%	80%	70%	60%	50%
WAC	0	10%	20%	30%	40%	50%
	**WAC-40R**	**WAC-30R**	**WAC-20R**	**WAC-10R**	**WAC**	
WAC-100R	40%	30%	20%	10%	0	
WAC	60%	70%	80%	90%	100%	

WAC means the total water extracts of charred areca nut, WAC-100R means the arecoline removed totally WAC.

**Table 2:** IC_50_ values of the different WAC-Rx samples on HaCaT cells (μg/mL).

	WAC-100R	WAC-90R	WAC-80R	WAC-70R	WAC-60R	WAC-50R
IC_50_	>400	>400	>400	>400	>400	>400
	**WAC-40R**	**WAC-30R**	**WAC-20R**	**WAC-10R**	**WAC**	
IC_50_	345.27	272.54	219.63	176.46	107.73	
