# Supplementary material for: Using the “target constituent removal combined with bioactivity assay” strategy to investigate the optimum arecoline content in charred areca nut
Source: Sci Rep. 2017 Jan 5;7:40278. doi: 10.1038/srep40278 (PMC5215198; doi:10.1038/srep40278)
Supplement: Supplementary Files [file srep40278-s1.doc]

**Using the “target constituent removal combined with bioactivity assay” strategy to investigate the optimum** **arecoline content in** **charred areca nut**

Wei Peng^1,2,†^, Yu-Jie Liu^1,†^, Mei-Bian Hu^1^, Dan Yan^1^, Yong-Xiang Gao^2^,*, Chun-Jie Wu ^1^,*

^1^ College of Pharmacy, Chengdu University of Traditional Chinese Medicine, Chengdu 610075, P.R. China.

^2^ College of Basic Medicine, Chengdu University of Traditional Chinese Medicine, Chengdu 610075, P.R. China.

*Correspondence author: Chun-Jie Wu;

Correspondence address: College of Pharmacy, Chengdu University of Traditional Chinese Medicine, No.1166 Liutai Avenue, Chengdu 610075, P.R. China;

Phone and Fax: +86-028-61801001;

E-mail: wucjcdtcm@163.com (Chun-Jie Wu).

^*^Co- correspondence author: Yong-Xiang Gao;

College of Basic Medicine, Chengdu University of Traditional Chinese Medicine, No.1166 Liutai Avenue, Chengdu 610075, P.R. China;

Phone and Fax: +86-028-61801001;

E-mail: gaoyxcdtcm@126.com (Yong-Xiang Gao).

^†^ These authors contributed equally to this manuscript.

**Results**

**Results of the HPLC assay of the total alkaloids of CAN extracted by CHCl_3_**

Results of the HPLC assays of the total alkaloids of CAN extracted by CHCl_3_ were shown in Figure S1. Our results showed that in the total alkaloids of CAN extracted by CHCl_3_, the main alkaloid is arecoline.


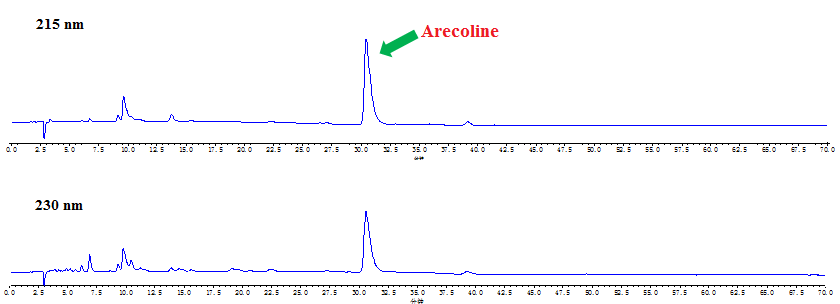


Figure **S1**. HPLC assays of the total alkaloids of CAN extracted by CHCl_3_.

**Results of the TLC assay of the total alkaloids of CAN extracted by CHCl_3_**

Results of the TLC assays of the total alkaloids of CAN extracted by CHCl_3_ were shown in Figure S2. We can find that there is no other obvious constituent interference in the same place of arecoline band. So, we think the PTLC could effective remove the arecoline and then prepare the WAC-100R sample.


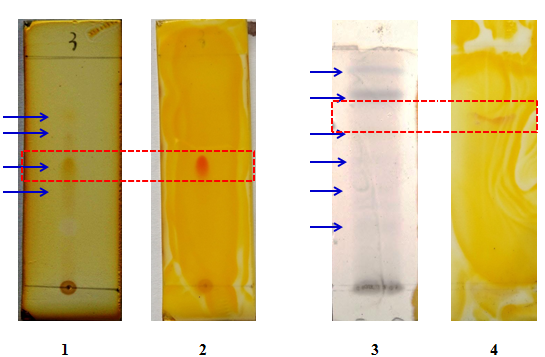


Figure S2. TLC assays of the total alkaloids of CAN extracted by CHCl_3_.

**Results of the arecoline contents determination by HPLC assay**

The contents of arecoline in charred areca nut (CAN) were determined and the results were shown in Table S1. The HPLC chromatograms of arecoline hydrobromide standard and the sample were shown in Figure S3. The results indicated that the average content of arecoline in CAN was 0.239 %.

**Table S1.** The contents of arecoline in CAN

| **No.** | **Contents of arecoline (%)** | **Average content (%)** |
| --- | --- | --- |
| 1 | 0.240 |  |
| 2 | 0.237 | 0.239 |
| 3 | 0.241 |  |

| 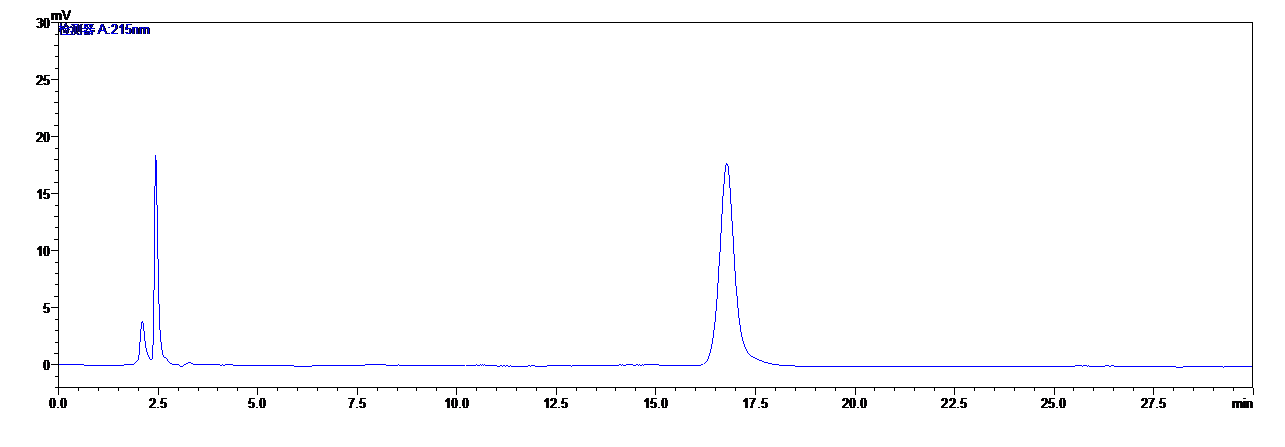  **A** |
| --- |
| 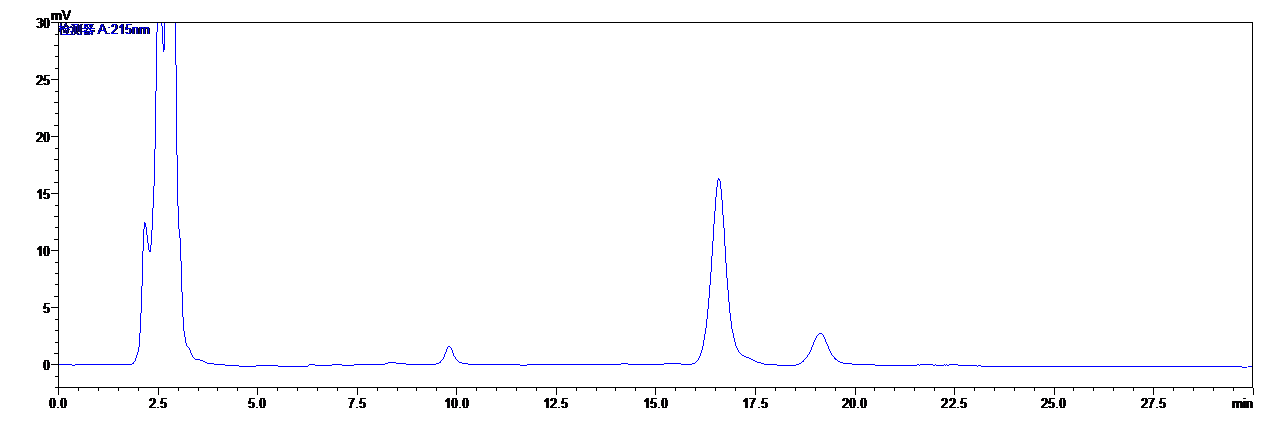  **B** |

**Figure S3.** The HPLC chromatograms of arecoline hydrobromide standard (A) and the sample (B).

**Materials and methods**

**HPLC assay of the arecoline contents in charred areca nut**

***Sample preparation***

The sample was powdered and sifted through the standard sieve (180μm ± 7.6μm). Approximately 0.3 g powder was accurately weighed and put into a conical flask. Then, the samples were extracted by reflux for three times (30 min for one time) with 50 mL ethyl ether [A volume of 3 mL carbonate buffer solution (1.91g sodium carbonate and 0.56g sodium bicarbonate dissolved in 100 mL water) was added to adjust the solvents pH value]. The ethyl ether solution was collected and 0.5 % phosphoric acid-water solution (1 mL) was added. A residue was obtained and dissolved by 50% acetonitrile, and filtered through a 0.22 μm filtration membrane before injection. Arecoline hydrobromide standard was dissolved with mobile phase as the standard solution.

**HPLC chromatographic conditions**

The HPLC chromatographic separation was performed using a gradient elution with a Nucleosil SA SCX (250 mm × 4.6 mm, 5um) column at 30 °C. The mobile phase was composed of 55% solvent A (acetonitrile) and 45% solvent B (0.2% phosphoric acid-water solution, the pH was adjusted to 3.8 with ammonium hydroxide) with a mobile flow rate of 1.0 mL/min. The detection wavelength was at 215 nm with the sample injection volume of 10.0μL.

**HPLC assay of the total alkaloids of CAN extracted by CHCl_3_**

HPLC assay was performed on an Agilent 1260 HPLC system with a CAPCELL PAK MG II S5 C_18_ chromatographic column (250 mm × 4.6 mm, i.d. 5 μm, Shiseido, Japan) at 215 and 230 nm, sample injection volume was 10 μL, and column temperature was set at 30 ºC. Separation was performed using gradient elution [acetonitrile (A) /0.1% aqueous formic acid (containing 0.1% sodium heptanesulfonate, B)] gradient at a flow rate of 1 mL/min. Samples were analyzed by using a gradient program as follows: 0 - 10 min, 92 % B; 10 - 40 min, 92 - 86% B; 40-70 min, 86 – 75 % B.

**TLC assay of the total alkaloids of CAN extracted by CHCl_3_**

All the TLC assays were performed by using the silica gel G TLC plates. For 1 and 2, the mobile phase was *n*-hexane - ethyl acetate – acetone – NH_3_.H_2_O (10:7:3:0.2); 1 and 2 were detected by iodine and bismuth potassium iodide reagents, respectively. For 3 and 4, the mobile phase was CHCl_3_ – acetone – NH_3_.H_2_O (7:3:0.2); 3 and 4 were detected by 10% sulfuric acid and bismuth potassium iodide reagents, respectively.
